# Multiparametrical diffusion weighted imaging for the detection of anaplastic transformation of low-grade gliomas

**DOI:** 10.1186/1470-7330-14-S1-S11

**Published:** 2014-10-09

**Authors:** MT Freitag, C Weber, K Maier-Hein, R Tarnawski, B Bobek-Billewicz, F Binczyck, J Polanska, B Stieltjes

**Affiliations:** 1 German Cancer Research Center, Quantitative Imaging-based Disease Characterization, Department of Radiology, Heidelberg, Germany; 2 German Cancer Research Center, Department of Medical Informatics, Heidelberg, Germany; 3 Maria Sklodowska-Curie Memorial Cancer Center and Institute of Oncology, Gliwice, Poland; 4 Silesian University of Technology, Data Mining Group, Gliwice, Poland

## Aim

The precise detection of anaplastic transformation in low-grade gliomas using magnetic resonance imaging (MRI) is impeded by postoperative changes in brain tissue. We tested the diagnostic value of diffusion tensor-derived axial diffusivity (AD), mean diffusivity (MD) (=apparent diffusion coefficient) and radial diffusivity (RD) maps in comparison to T1w and T2w sequences.

## Methods

The study was approved by the local ethics committee. Forty-seven patients with histopathologically proven low-grade glioma II° were included, 28 were stable and 19 patients had post-surgical anaplastic transformation. All patients underwent pre-operative MRI, surgery and subsequent post-operative MRI follow-ups at 1.5T including T1w, T2w sequences and a DTI-protocol. The scalar indices AD, MD and RD were calculated voxel-by- voxel for all patients from the tensor eigenvalues and the minimum value within a gross tumour segmentation was extracted using MITK-Diffusion, respectively.

## Results

Hypointense clusters were seen in every patient with anaplastic transformation in the DTI maps with best contrast-to-noise in AD. In 65% of patients with anaplastic transformation, these clusters were noticed at the same time when compared to contrast enhancement. In 35% of patients, hypointense changes were visible in AD maps in examinations prior to the initial contrast enhancement. ADmin showed best combined sensitivity/specificity (94.4%/89.7%, AUC 0.96) to indicate transformation.

## Conclusion

AD maps provide additional essential information for anaplastic transformation of low-grade gliomas after resection and indicate the progress at the same time or earlier when compared to T1w-CE. We conclude that it is advisable to use a DTI-protocol instead of standard diffusion-weighted imaging for neuro-oncological exams.

